# Optimizing Stem Length in Conversion Total Hip Arthroplasty: An Expanded Finite Element Analysis

**DOI:** 10.3390/jcm14041141

**Published:** 2025-02-10

**Authors:** Koshiro Shimasaki, Tomofumi Nishino, Tomohiro Yoshizawa, Ryunosuke Watanabe, Fumi Hirose, Shota Yasunaga, Hajime Mishima

**Affiliations:** Department of Orthopaedic Surgery, Institute of Medicine, University of Tsukuba, 1-1-1, Tennodai, Tsukuba 305-8575, Japan; koshiro19881020@tsukuba-seikei.jp (K.S.); tyoshizawa@tsukuba-seikei.jp (T.Y.); ryuwatanabe@tsukuba-seikei.jp (R.W.); f.ochiai.0023@tsukuba-seikei.jp (F.H.); syasunaga@tsukuba-seikei.jp (S.Y.); hmishima@tsukuba-seikei.jp (H.M.)

**Keywords:** conversion total hip arthroplasty (cTHA), finite element analysis (FEA), stress concentration, periprosthetic fracture, stem length

## Abstract

**Background/Objectives**: Stress concentration around distal screw-removal holes confers a major risk for periprosthetic fractures following conversion total hip arthroplasty (cTHA) for intertrochanteric femoral fractures. Optimal stem-selection criteria and guidelines for cTHA can improve clinical outcomes. We determined the influence of the cementless stem length on the stress distribution around distal screw-removal holes. **Methods**: For the finite element analysis, institutional data from preoperative CT scans of contralateral femurs of patients who underwent THA were used. To replicate the post-nail-removal state, we used 3D registration of standard triangulated language data of the intramedullary nail as an unused material to simulate distal screw-removal holes, located 135 mm from the proximal end of the intramedullary nail. Cementless stems of 130, 140, 150, and 160 mm were individually registered using STL data, and cTHA models were constructed accordingly. Using simulations under load conditions representing normal walking and stair climbing, the mean and maximum equivalent stress values around the distal screw-removal holes were calculated. For multiple comparisons, repeated-measures ANOVA with Bonferroni correction was employed. **Results**: Compared to the 130 mm stem, the 150 mm and 160 mm stems similarly reduced the maximum equivalent stress around the distal screw-removal holes, although the 140 mm stem showed no significant difference with other stems. **Conclusions**: A ≥150 mm stem length reliably mitigated stress concentration around distal screw-removal holes post-cTHA; it is the optimal choice for balancing effectiveness and risk of complications and may contribute to improved long-term clinical outcomes. This study provides practical evidence for stem selection in cTHA and offers valuable insights for future treatment guidelines.

## 1. Introduction

Among older adults, intertrochanteric femoral fractures are common, and unstable types are frequently treated surgically using intramedullary nails [1,2]. However, patient-related factors, such as bone fragility, as well as surgical factors, including insufficient reduction or improper implant positioning, may lead to complications, such as femoral head cut-out, cut-through, nonunion, or pseudoarthrosis. Furthermore, conditions such as osteoarthritis or necrosis of the femoral head may constitute indications for salvage surgery, including conversion total hip arthroplasty (cTHA) [3,4,5,6].

Compared to primary total hip arthroplasty (pTHA), cTHA involves greater complexity, is associated with higher perioperative complication rates, and generally yields inferior clinical outcomes [7,8,9,10,11,12]. For both cTHA and pTHA, periprosthetic fractures are among the most common perioperative complications, second only to dislocation [13,14,15]. Periprosthetic fractures not only significantly affect the activities of daily living and quality of life of patients but also constitute the most expensive perioperative complication, with an approximate cost of USD 4000 per case [16]. In Japan, with the increasing proportion of the older population, it is estimated that the incidence of intertrochanteric fractures and the consequent need for cTHA will increase. Therefore, the prevention and management of complications constitute critical challenges for orthopedic surgeons.

Among cTHA-associated periprosthetic fractures, stress concentration following stem insertion may cause fractures around distal screw-removal holes [8,17,18,19,20,21]. These fractures frequently occur with minor trauma or without a clear injury mechanism [11,22]. Despite numerous studies on stem selection in cTHA, no significant difference in clinical outcomes, including perioperative complications, have been demonstrated, and, therefore, stem selection remains largely dependent on the surgeon’s experience and preference [23,24,25,26,27,28,29,30,31,32]. Although cemented stems are sometimes selected to address bone fragility, their use requires considerable expertise, including troubleshooting. Furthermore, the presence of multiple cortical defects or bone voids from prior surgery may hamper adequate cement pressurization, which generates concerns regarding the bone cement implantation syndrome. Thus, a considerable proportion of orthopedic surgeons prefer to use cementless stems [24,25,26].

The tip of a standard stem frequently aligns near the distal screw-removal hole, which potentially exacerbates stress concentration and increases the risk of periprosthetic fractures. Consequently, revision long stems are frequently selected [14,24,25,26,27,28,33], and their selection is supported by biomechanical studies, which suggest that, to ensure stability and stress distribution, fractures and large cortical bone defects should be bridged by implants that extend across at least twice the femoral diameter (approximately 40 mm) [34,35,36].

Nonetheless, it is unclear whether small cortical bone defects, such as distal screw-removal holes, significantly weaken the femoral diaphysis under lower-limb loading. Haidukewych et al. recommended intraoperative filling of cortical bone defects caused by prior screws whenever feasible, as these defects may compromise cement pressurization for cemented stems or constitute stress-concentration sites for cementless stems, and thereby increase the risk of femoral fractures [14]. However, if screw-removal holes are, indeed, a source of weakness, then the necessary bridging length remains undefined; thus, the effect of stem length on stress distribution around screw-removal holes is unclear.

In our previous study [37], we used standard triangulated language (STL) data for three types of cementless stems—120 mm (0 mm bridging length relative to the distal screw-removal hole), 130 mm (10 mm bridging), and 160 mm (40 mm bridging)—to compare and evaluate stress distribution using finite element analysis (FEA). Compared to the insertion of 120 mm and 130 mm stems, the insertion of a 160 mm stem, which was designed as a revision long stem, significantly reduced the maximum stress on the distal screw-removal hole. Therefore, we concluded that to reliably avoid stress concentration at the distal screw-removal hole when employing a cementless stem in clinical practice, a revision long stem is beneficial. However, the previous study did not clarify whether the 160 mm stem represented the minimum necessary length to avoid stress concentration at the distal screw-removal hole, nor elucidate whether longer stems might be excessive. Additionally, compared to standard stems, the use of long stems in cTHA is associated with significantly longer surgical times, increased blood loss, and higher rates of intraoperative fractures [38]. Thus, if similar effects can be achieved with shorter stems, then this could potentially reduce perioperative risks.

In the present study, we aimed to undertake a detailed investigation of the influence of the cementless stem length on the stress distribution around distal screw-removal holes and identify the minimum required length of cementless stems. We believe that refining the selection of optimal stems for cTHA will mitigate the risks of intraoperative and perioperative complications.

To further investigate the optimal stem length for cTHA, we extended our previous study [37] by incorporating the following two additional stem lengths: 140 and 150 mm (bridging length: 20 and 30 mm, respectively). Using FEA simulations, we analyzed the maximal stress around the distal screw-removal hole and conducted statistical evaluations to identify the necessary and sufficient stem length for cTHA.

Based on prior biomechanical studies [34,35,36] and our previous findings [37], we hypothesized that, to significantly reduce the maximal stress around the distal screw-removal hole, the optimal stem length should lie between 130 and 160 mm. Considering the lower axial stability demands for smaller defects compared to fractures or large cortical voids, we speculated that the sufficient bridging length would be less than 40 mm. This study used FEA to elucidate the optimal length of cementless stems in cTHA following intertrochanteric fractures.

## 2. Materials and Methods

### 2.1. Study Setting and Design

This analytical observational study utilized computed tomography (CT) data obtained from a single institution from October 2021 to September 2024. Participants provided informed consent for the analysis and publication of these data. The Ethics Committee of the University of Tsukuba Hospital approved this analytical observational study (approval code: H27-041). We used FEA to determine the optimal length of cementless stems for cTHA following intertrochanteric femoral fractures. Simulations were conducted using pre-existing patient CT data and standard triangulated language (STL) data of femoral stems to analyze femoral stress distribution. This expanded study builds upon our earlier work that was aimed at identifying the optimal stem length for cTHA following intertrochanteric femoral fractures.

### 2.2. Study Samples

The required sample size was calculated using G*Power (version 3.1.9.7, University of Düsseldorf, Germany). Assuming an effect size of 0.25, significance level of 0.05, statistical power of 0.8, and four groups, the minimum sample size was determined to be 24. Based on this calculation, we utilized the same 30 femoral CT datasets that were utilized in our previous study.

**Figure 1:**
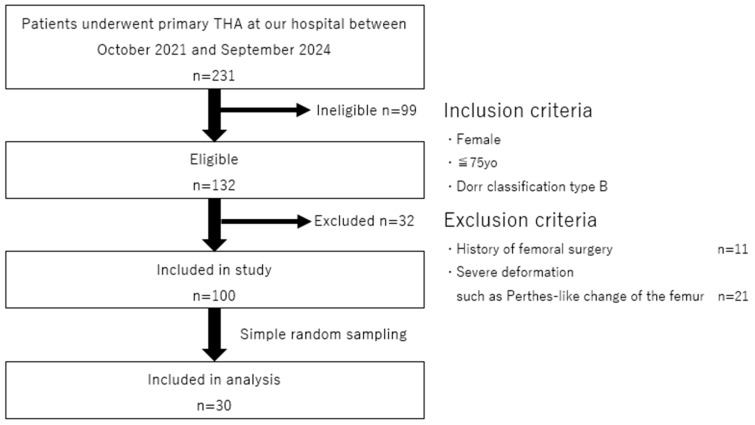
Flowchart depicting the patient screening and participant selection processes in this study.

From among 231 patients who underwent pTHA at our institution between October 2021 and September 2024, we identified 132 female patients aged ≤75 years with Dorr type B [39] femoral morphology. This selection was based on the fact that most cases of femoral trochanteric fractures occur in elderly women and the need to minimize variations in the shape of the femoral medullary canal among patients for a more consistent analysis. Of these, 11 patients with a history of femoral surgery and 21 with significant deformities, such as Perthes-like deformities, were excluded. From the remaining 100 eligible cases, 30 cases were randomly selected using a simple random sampling method, and CT data of the contralateral femurs of these patients were used for analysis (Figure 1).

### 2.3. Finite Element Analysis

#### 2.3.1. Software and Modeling

We performed FEA using MECHANICAL FINDER (MF, version 13.0, Extended Edition, Research Center of Computational Mechanics, Tokyo, Japan). First, 3D femoral models were generated by importing the contralateral femoral CT data into MF. Next, to replicate the femur after hardware removal, the STL data from a Trochanteric Fixation Nail Advanced Proximal Femoral Nailing System (TFNA, Depuy Synthes, Raynham, MA, USA; nail: φ10 mm × 200 mm/130°; blade: φ10.35 mm; distal screw: φ5 mm) were registered and replaced with voids by setting the material property to “unused material”. The distal screw has a diameter of 5 mm and, in static fixation, is designed to be positioned 135 mm from the proximal end of the intramedullary nail, which was inserted into the 3D femur model with the tip apex distance set to less than 20 mm. This model was referred to as the “extraction model”.

**Figure 2:**
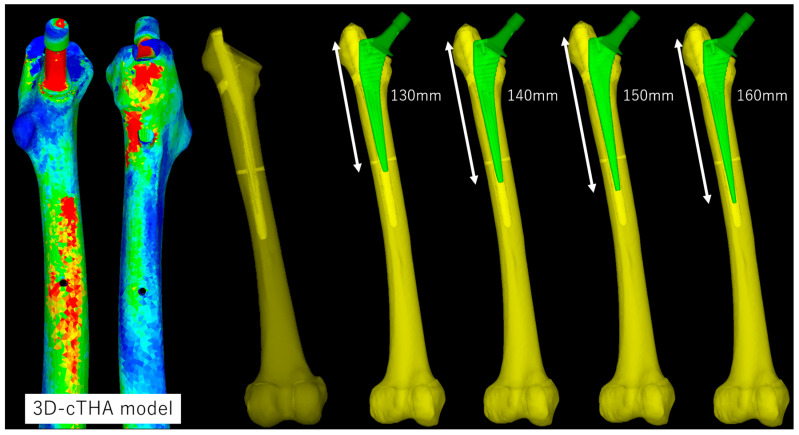
The 3D-cTHA model was constructed by inserting a stem into a 3D femoral model after the removal of the intramedullary nail. Four stem lengths (130, 140, 150, and 160 mm) were used, with each stem bridging the distal screw-removal hole by 10, 20, 30, and 40 mm, respectively.

**Figure 3:**
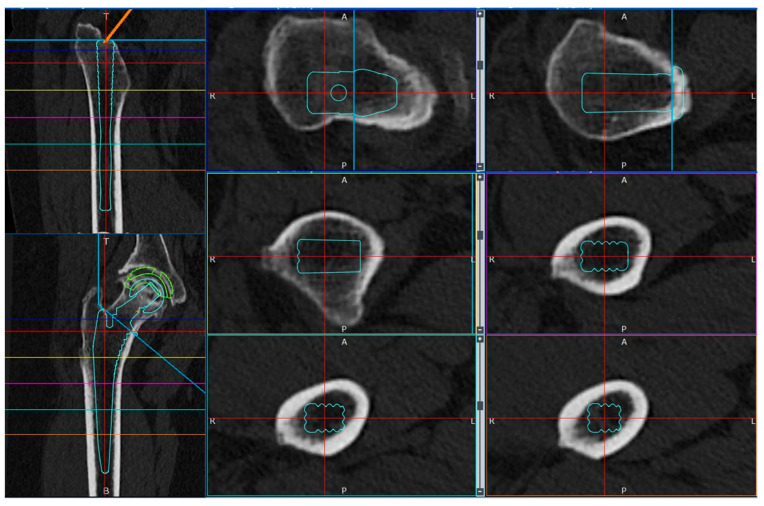
Preoperative planning for cTHA was performed using the 3D software ZedHip (version 17.0.0).

Subsequently, STL data of the cementless stem with full circumferential hydroxyapatite coating, the Universia stem (#11, high offset; Teijin Nakashima Medical, Okayama, Japan) [40] were inserted into the extraction model to create a cTHA model (Figure 2). Neck osteotomy and stem positioning were planned using ZedHip (version 17.0.0, Lexi Co., Ltd., Tokyo, Japan) and faithfully reproduced in MF (Figure 3). The following four stem lengths were defined (Figure 2): (1) 130 mm (bridge length: 10 mm); (2) 140 mm (20 mm); (3) 150 mm (30 mm); and (4) 160 mm (40 mm). Among these, only the 130 mm stem is clinically available; the other lengths are virtual stems designed by editing the STL data for simulation purposes.

#### 2.3.2. Material Parameters

Tetrahedral elements with four nodes were used as solid elements. A shell element with a 0.001 mm thickness was applied to the bone surface because it does not affect structural strength. Mesh sizes were determined based on a mesh convergence test from our previous study; accordingly, we selected a size of 1–2 mm to balance computational efficiency and accuracy.

The bone material properties were modeled as inhomogeneous, and the Young’s modulus was derived from bone mineral density (BMD) calculated using Hounsfield unit (HU) values, based on a linear relationship [41,42]. Keyak’s predictive conversion formula was applied for the estimation [43]. The Poisson’s ratio was set to 0.40. The titanium alloy (Ti-6Al-4V) used for the stem was modeled as a homogeneous material with a Young’s modulus of 109 GPa and a Poisson’s ratio of 0.28 (Table 1) [41,43].

#### 2.3.3. Loading and Boundary Conditions

**Figure 4:**
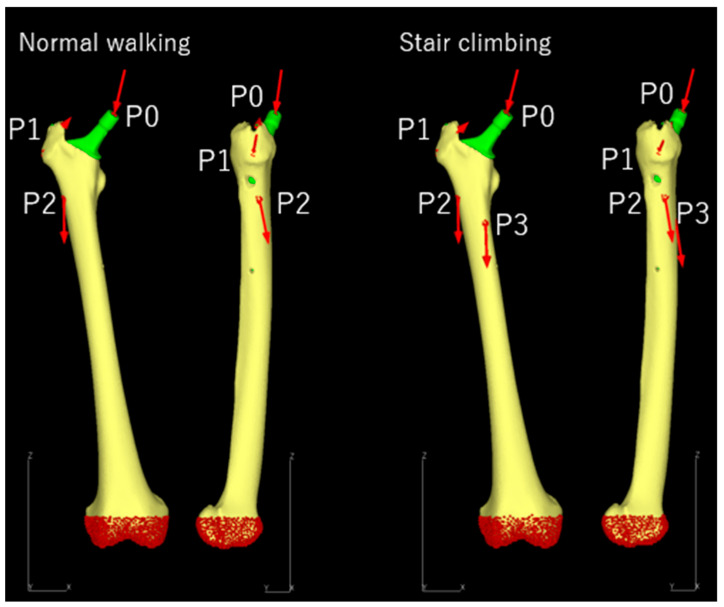
Load conditions: The points of the load application and fixation sites for normal walking and stair climbing are shown. P0 represents the hip-joint contact point; P1 is the point of action of the combined forces from the abductor muscles and iliotibial ligament; P2 is the point of action of the vastus lateralis; and P3 is the point of action of the vastus medialis. In both conditions, the distal femur was fully modeled (highlighted in red).

Simulations were performed under maximum loading conditions during “normal walking” and “stair climbing”, as reported in previous studies [44,45]. The load vectors, magnitude, and directions were determined based on patient body weight (Table 2, Figure 4). The distal femur was fully constrained, and a friction coefficient of 0.49 was applied to the contact surface between the femur and stem [46].

#### 2.3.4. Static Structural Analysis

Linear static analysis was performed with gradually increasing loads.

### 2.4. Data Collection and Candidate Predictors

The demographic data, including age, height, weight, body mass index (BMI), femoral neck BMD, and the Canal Flare Index (CFI) [47], of the participants were retrospectively collected from medical records. Preoperative measurements were used for age, height, weight, and BMI, while BMD was assessed using Hologic Discovery A (Hologic Inc., Bedford, MA, USA). Parameters such as the stem anteversion angle, bridge length, and distal screw length were measured on the constructed cTHA models.

The stem anteversion angle was defined as the angle between the stem neck and the posterior condylar axis. Measurements were conducted three times under identical conditions by the same investigator (K.S.), and the mean values were used to minimize variability and enhance reliability. Data were uniformly expressed as the mean ± standard deviation (SD).

### 2.5. Outcomes

**Figure 5:**
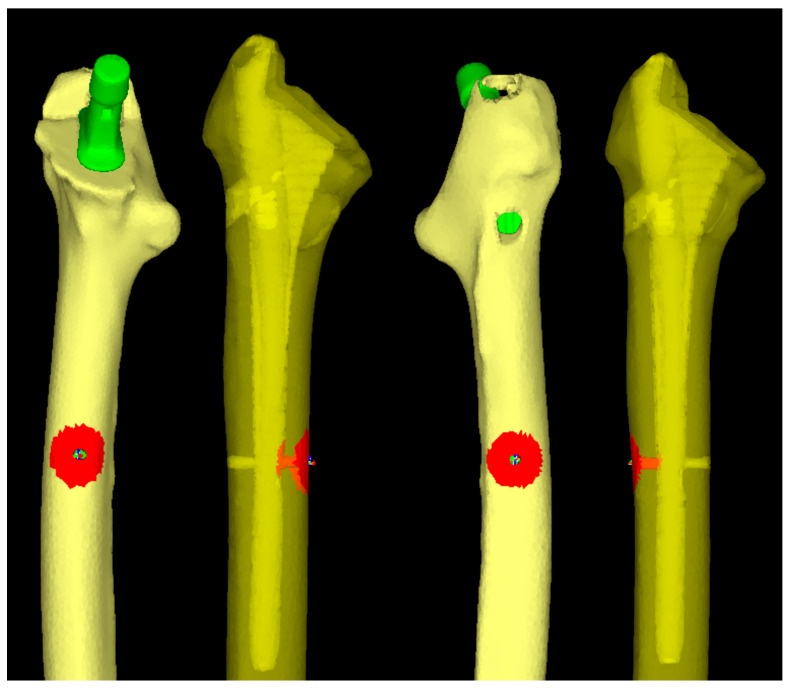
The region of interest for stress measurement was defined as the shell elements on the bone surface in a spherical area within a 10 mm radius centered around the distal screw-removal hole (highlighted in red).

Simulations were performed under “normal walking” and “stair climbing” conditions for stems with 130, 140, 150, and 160 mm lengths. The mean and maximum equivalent stress values on the inner and outer surfaces around the distal screw-removal hole were calculated. The analysis focused on shell elements within a 10 mm radius sphere centered on the distal screw-removal hole (Figure 5). Data are reported as the mean value with the 95% confidence interval (95% CI).

### 2.6. Statistical Analysis

Statistical analysis was conducted using SPSS Statistics (version 29.0.2.0, IBM Corp., Armonk, NY, USA). Normality was assessed with the Shapiro–Wilk test. A significance level of α = 0.05 was adopted. If the data were normally distributed (*p* > 0.05), intergroup comparisons were undertaken using parametric tests.

## 3. Results

The characteristics of the participants and the 3D-cTHA model constructed in this study are summarized in Table 3.

**Figure 6:**
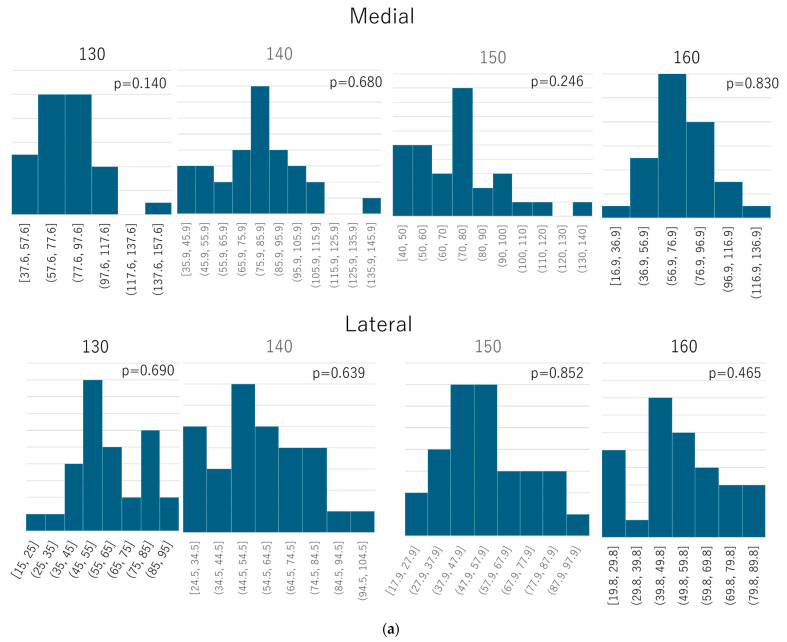

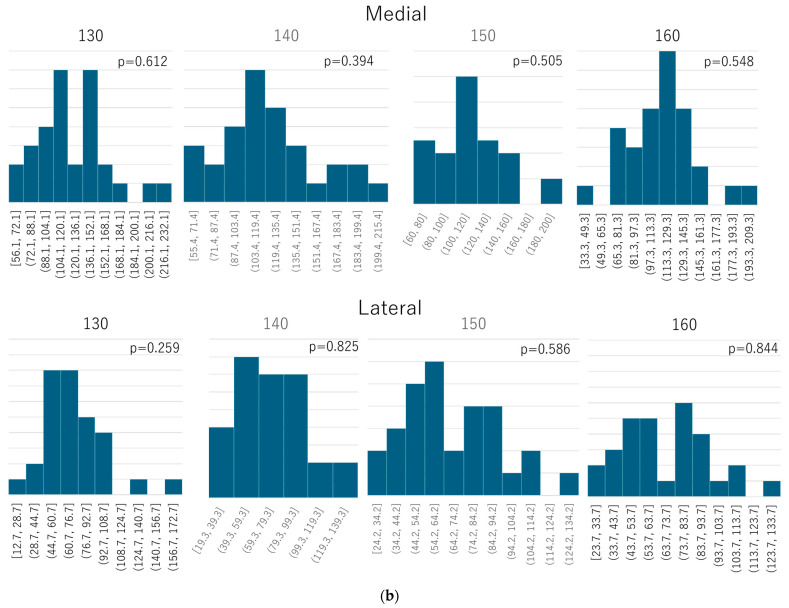
Histograms of normality tests performed using the Shapiro–Wilk test: (**a**) normal walking; (**b**) stair climbing. The data under both “normal walking” and “stair climbing” conditions followed a normal distribution.

The maximum equivalent stress at the medial and lateral regions of the distal screw-removal holes for the four groups (130, 140, 150, and 160 mm) under both “normal walking” and “stair climbing” conditions followed a normal distribution. The results of the Shapiro–Wilk test were as follows:

- Normal walking—medial: 130 mm: W = 0.947, *p* = 0.140; 140 mm: W = 0.975, *p* = 0.680; 150 mm: W = 0.956, *p* = 0.246; 160 mm: W = 0.980, *p* = 0.830;
- Normal walking—lateral: 130 mm: W = 0.975, *p* = 0.690; 140 mm: W = 0.974, *p* = 0.639; 150 mm: W = 0.981, *p* = 0.852; 160 mm: W = 0.967, *p* = 0.465;
- Stair climbing—medial: 130 mm: W = 0.973, *p* = 0.612; 140 mm: W = 0.964, *p* = 0.394; 150 mm: W = 0.969, *p* = 0.505; 160 mm: W = 0.970, *p* = 0.548;
- Stair climbing—lateral: 130 mm: W = 0.957, *p* = 0.259; 140 mm: W = 0.980, *p* = 0.825; 150 mm: W = 0.972, *p* = 0.586; 160 mm: W = 0.981, *p* = 0.844 (Figure 6).

Based on these results, repeated-measures analysis of variance (ANOVA) was selected for comparison of the four groups using paired data. Post hoc multiple comparisons were performed with Bonferroni correction to assess the differences among all group combinations.

### 3.1. Mean Equivalent Stress Around Distal Screw-Removal Holes

**Figure 7:**
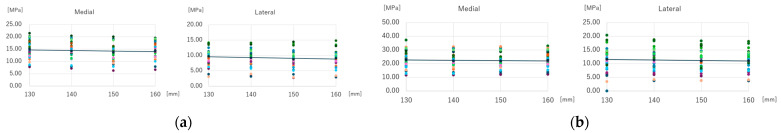
Comparison of the means of the von Mises stress: (**a**) normal walking; (**b**) stair climbing. In both conditions, the average stress around the distal screw-removal hole decreased as the stem length increased.

Scatterplots of the mean equivalent stress at the distal screw-removal holes and their trendlines are shown in Figure 7. Under both “normal walking” and “stair climbing” conditions, the mean equivalent stress decreased as the stem length increased.

### 3.2. Maximum Equivalent Stress Around Distal Screw-Removal Holes

#### 3.2.1. Normal Walking Condition

##### Comparison Across the Four Groups

In the ANOVA on both the medial (F(3, 87) = 6.263, *p* < 0.001, η^2^ = 0.18) and lateral sides (F(3, 87) = 7.752, *p* < 0.001, η^2^ = 0.21; Table 4), significant main effects were observed.

##### Post Hoc Multiple Comparisons

Compared to the 130 mm group, we found significant intergroup differences for the 150 mm and 160 mm groups on both the medial and lateral sides (130 mm vs. 150 mm: medial, *p* = 0.035; lateral, *p* = 0.006; 130 mm vs. 160 mm: medial, *p* = 0.006; lateral, *p* = 0.007). On the medial side, the stress values for the 150 mm and 160 mm groups were lower by a mean value of 6.300 MPa (95% CI: −12.283, −0.317) and 6.395 MPa (95% CI: −11.333, −1.457), respectively, compared to the 130 mm group. On the lateral side, the stress values for the 150 mm and 160 mm groups were lower by an average of 5.190 MPa (95% CI: −9.223, −1.157) and 5.600 MPa (95% CI: −9.989, −1.211), respectively.

**Figure 8:**
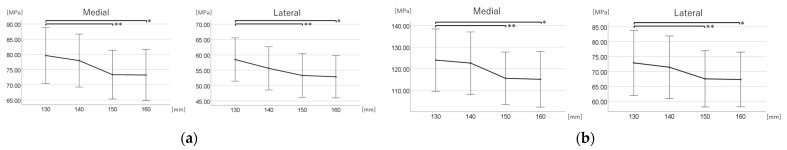
Comparison of the maximum equivalent stress: (**a**) normal walking; (**b**) stair climbing. In both conditions, the following results were observed: (1) compared to the 130 mm stem, the stress was significantly lower for the 150 mm and 160 mm stems; (2) no significant difference was observed between the 150 mm and 160 mm stems; (3) compared to the other stem lengths, the 140 mm stem showed no significant difference. * and ** indicate significant differences between the groups.

No significant differences were observed between the 140 mm group and any other group (140 mm vs. 130 mm: medial, *p* > 0.99; lateral, *p* = 0.210; vs. 150 mm: medial, *p* = 0.155; lateral, *p* = 0.384; vs. 160 mm: medial, *p* = 0.119; lateral, *p* = 0.191) or between the 150 mm and 160 mm groups (medial, *p* > 0.99; lateral, *p* > 0.99) (Figure 8).

#### 3.2.2. Stair Climbing Condition

##### Comparison Across the Four Groups

Significant main effects (Table 4) were observed in the ANOVA for both the medial (F(3, 87) = 3.495, *p* = 0.019, η^2^ = 0.11) and lateral sides (F(3, 87) = 4.822, *p* = 0.004, η^2^ = 0.14).

##### Post Hoc Multiple Comparisons

Significant differences were found between the 130 mm group and both the 150 mm and 160 mm groups on both the medial and lateral sides (130 mm vs. 150 mm: medial, *p* = 0.036; lateral, *p* = 0.010; 130 mm vs. 160 mm: medial, *p* = 0.007; lateral, *p* = 0.019). On the medial side, the stress values for the 150 mm and 160 mm groups were lower by an average of 8.430 MPa (95% CI: −16.474, −0.386) and 8.870 MPa (95% CI: −15.870, −1.870), respectively, compared to the 130 mm group. On the lateral side, the stress values for the 150 mm and 160 mm groups were lower by an average of 5.350 MPa (95% CI: −9.710, −0.990) and 5.557 MPa (95% CI: −10.435, −0.678), respectively.

We found no significant differences between the 140 mm group and any other group (140 mm vs. 130 mm: medial and lateral, *p* > 0.99; vs. 150 mm: medial, *p* = 0.488; lateral, *p* = 0.393; vs. 160 mm: medial, *p* = 0.548; lateral, *p* = 0.433), nor between the 150 mm and 160 mm groups (medial and lateral, *p* > 0.99) (Figure 8).

## 4. Discussion

We conducted an expanded study by utilizing FEA to investigate the optimal stem length for cTHA following intertrochanteric femoral fractures. This study builds on the methodology and design of a previous study [37]. With an increasing stem length on both the medial and lateral sides, the average equivalent stress gradually decreased at the distal screw-removal holes. The maximum equivalent stress values were as follows:

I. Compared to the 130 mm stem, the 150 mm and 160 mm stems were associated with significantly lower stress;
II. There was no significant difference between the 150 mm and 160 mm stems in the stress reduction;
III. The 140 mm stem showed no significant difference compared to any other stem length.

For fractures and large bone defects, it is generally recommended to bridge gaps with implants, such as stems or intramedullary nails, that have at least twice the bone diameter (approximately 40 mm) [34,35,36]. Moreover, this principle underpins the treatment strategies for other conditions that require stem bridging, such as Vancouver type B2 or B3 fractures.

Despite the lack of an established consensus regarding the management of relatively small cortical defects, such as screw-removal holes, some reports related to the management of cTHA have been published. Unlike intramedullary nails, reports on extramedullary fixation devices, such as nail plates, suggest that fractures were not induced despite no extended stem bridging over the screw-removal holes [48]. Postmortem studies have demonstrated that bridging lengths of 1.5 times the femoral diameter minimized stress at screw-removal holes [49].

Biomechanical studies on bone hole strength have been reported. Those using animal bones revealed that drill holes 3–4 mm in diameter or cortical defects exceeding 20% of the bone diameter significantly reduced the energy-absorption capacity, although the torsional strength was not greatly affected [50,51,52,53].

In this study, the distal screw-removal holes correspond to approximately 20% bicortical defects, as the distal screw diameter was 5 mm and the femoral diameter was 25.7 ± 1.7 mm. Thus, these areas may be structurally vulnerable during load transmission. To avoid stress concentration and prevent fractures at distal screw-removal holes, it is recommended that a stem of sufficient length is used for cTHA.

Our expanded study revealed that 150 mm (bridging length: 30 mm) or 160 mm (bridging length: 40 mm) stems are desirable to reduce stress at distal screw-removal holes. Although stems longer than 160 mm might provide greater stress distribution, this remains unclear. Furthermore, longer stems are associated with an increased operative time, intraoperative bleeding, and perioperative complications [41]. Considering that it achieves a similar stress distribution as the 160 mm stem, the 150 mm stem is the preferred option.

The 140 mm stem represents a middle-ground approach. When the stem length increases and, consequently, the bridging length over the bone hole extends, the maximum equivalent stress decreases. Although the 140 mm stem, with a 20 mm bridging length, reduced stress, the extent of the stress reduction was likely insufficient to achieve statistical significance. Although shorter stems preserve bone stock and promote physiological stress distribution, they may increase stress on weak bones and thereby confer a higher risk of peri-stem fractures in osteoporotic patients. Conversely, longer stems reduce localized stress concentration but may cause proximal stress shielding and, accordingly, lead to proximal femoral bone resorption [54,55,56]. In clinical practice, especially among Japanese individuals with a smaller build and curved femoral shapes, achieving a sufficient stem length may not always be feasible. A 140 mm stem, therefore, could offer a balanced solution for minimizing perioperative complication risks and maintaining adequate strength by avoiding the use of excessively long stems that might be incompatible with the patient’s femoral anatomy or bone fragility. Depending on the case, this trade-off might represent a well-balanced and viable option for improved clinical outcomes. Depending on the type of intramedullary nail used in the initial surgery, the position of the distal screw-removal hole can vary slightly. In the comparison of the distal screw-removal hole positions under static fixation for the major intramedullary nails that are currently used for intertrochanteric femoral fractures, the TFNA (Depuy Synthes, Raynham, MA, USA) and InterTAN (Smith & Nephew, Memphis, TN, USA) both had distal screw-removal holes located 135 mm from the proximal end of the intramedullary nail. In contrast, for the Gamma 3 nail (Stryker, Mahwah, NJ, USA), the distal screw-removal hole is positioned 140 mm from the proximal end of the intramedullary nail. Consequently, the stem length required to ensure sufficient bridging length in cTHA is expected to be longer, and this warrants careful consideration.

In any case, when selecting from the stem lengths available for clinical use, cementless stems inevitably require the use of revision long stems, leaving limited options. Moving forward, it is hoped that the development of stems of more appropriate lengths tailored to individual patients, from the perspective of stress distribution, will provide orthopedic surgeons with greater flexibility in their choices.

Structurally weak regions, such as screw-removal holes, are prone to stress concentration and increase the fracture risk. When stems of insufficient length are used, the placement of the stem tip near the screw-removal holes may amplify stress concentration through a hinge effect.

In general, inserting a stem with greater rigidity than the femur facilitates broader stress distribution via the stem. Longer stems, being more rigid, are likely to distribute stress more effectively and reduce localized stress at screw-removal holes. Furthermore, stems may physically reinforce the structural weakness at screw-removal holes.

Significant fractures or cortical bone defects critically affect bone strength and require robust implant bridging for ensuring axial stability, whereas smaller defects, such as screw-removal holes, may have a more limited impact. In cTHA, the primary purpose of bridging is to reinforce strength and stiffness in response to cortical bone deficiencies and thereby reduce stress. It is presumed that the necessary and sufficient bridging length may be shorter compared to the length required for fractures or significant cortical bone defects. In this study, we found that a stem length of 150 mm was the sufficient length required to avoid stress concentration at the distal screw-removal hole, and it corresponds to a bridging length of 30 mm, or 1.2 times the femoral diameter.

The number of patients with intertrochanteric fractures is expected to increase with population aging and could thereby increase the demand for cTHA; this highlights the importance of strategies to address postoperative complications. Among these, periprosthetic fractures—particularly around distal screw-removal holes—represent significant complications, with surgical factors, such as stress concentration, playing a potential role.

Despite the empirical use of long stems by many orthopedic surgeons, no prior study has rigorously examined the relationship between stem length and stress distribution around screw-removal holes. A previous study [37] demonstrated via FEA that long stems significantly reduced the maximum stress at distal screw-removal holes. The present expanded study further examined stem lengths in greater detail by employing repeated measurements to reduce data variability and improve statistical power, thereby minimizing the required sample size. Although additional simulations and statistical analyses were necessary, compared to strain gauge methods or thermoelastic stress analysis, this study achieved cost and time efficiencies. Importantly, FEA enabled detailed analysis of femoral stresses, including those within the screw-removal holes—an area that is inaccessible to analysis using other methods.

The strength of this research is that the results provide new insights into the optimal stem length in cTHA and offers valuable data to guide orthopedic surgeons in the selection of appropriate stems.

Nonetheless, the limitations of this study and future perspectives warrant discussion. First, similar to our previous study, this research was conducted under idealized conditions, which do not fully replicate in vivo environments, including bone heterogeneity and dynamic load conditions. Using MF software (version 13.0), we incorporated patient-specific bone microstructures by estimating bone density from CT values. Simulating maximal daily living loads with muscle dynamics aimed to approximate real-world scenarios. Nevertheless, the CT scanner settings significantly impacted the FEA model’s accuracy by necessitating a balance between the resolution and computational efficiency. Second, to simplify the model, this study did not replicate fracture lines, callus formation, or bone sclerosis around intramedullary nails and screw-removal holes. Although these factors could be included using post-nail-removal CT data, such cases are rare, which makes adequate sample collection challenging. We addressed this by replacing nail properties with the “unused material” settings in MF software to simulate post-removal conditions. Third, the position of the distal screw hole, femoral neck osteotomy, and stem depth can also be variables. Generally, in osteosynthesis for femoral trochanteric fractures, an intramedullary nail that matches the femoral neck-shaft angle is selected and inserted with the goal of achieving a tip-apex distance (TAD) of within 20 mm. Therefore, when using the same intramedullary nail, there is minimal variation in the position of the distal screw hole, even among different patients. However, as mentioned in the text, the insertion position of the distal screw may vary depending on the type of intramedullary nail, which requires careful consideration. Fourth, in this study, to ensure that the prosthetic femoral head accurately replicates the center of the patient’s original femoral head, the level of the femoral neck osteotomy was determined and the stem depth was adjusted for each patient using a 3D preoperative planning software in accordance with actual clinical procedures. Regarding the removal holes of the intramedullary nail, their impact is considered minimal since they do not vary significantly depending on the type of nail.

This study focused solely on stem length and excluded factors such as stem diameter, shape, and fixation concepts. Although the stem size was standardized to #11, variations in patient-specific femoral canal shapes may have influenced the results. Future studies should consider these parameters. Thus, although this study focused on the evaluation of stem length, the evident approach to further investigations would involve the generation of multiple hypothetical stem CAD models while incorporating variations in parameters, such as diameter, shape, and fixation concepts, followed by the validation of each of these models. Alternatively, validations would need to be conducted for each variation in the available stems.

Finally, FEA remains a simulation-based approach that requires experimental validation. Subsequent research should include physical models and clinical trials to complement and verify the simulation findings. Further research on experimental validation using thermoelastic stress analysis on a simulated femur to extend the insights from this study is warranted. Moreover, comparative studies with cemented stems in osteoporotic models may be valuable.

## 5. Conclusions

Preoperative simulation using FEA is highly useful for determining the optimal stem length in cTHA following intertrochanteric femoral fractures. When performing cTHA with a cementless stem and a full circumferential hydroxyapatite coating such as Universia stem, following intramedullary nailing for intertrochanteric femoral fractures, a bridging length of at least 30 mm is desirable to avoid stress concentration at the distal screw-removal hole. In this study, a bridging length of 30 mm corresponded to approximately 1.2 times the femoral diameter. This length is particularly optimal when balancing the minimization of the risk of complications and maximizing the effectiveness, and potentially contributes to improved long-term clinical outcomes. Therefore, in clinical practice, the use of revision long stems is recommended.

## Figures and Tables

**Table 1:** Material parameters.

	Material	Young’s Modulus [GPa]	Poisson’s Ratio
Femoral bone	Heterogeneous model	Keyak et al. [43]	0.40
Stem	Titanium alloy (Ti-6Al-4V)	109	0.28

**Table 2:** Loading conditions during normal walking and stair climbing.

**Normal Walking**					
**Force**	**X (N)**	**Y (N)**	**Z (N)**	**Acting Point**	**%**
Hip contact	Lt. 54.0/Rt. −54.0	32.8	−229.2	P0	238
ABD	Lt. 58.0/Rt. −58.0	4.3	86.5	P1	104
TFL-P	Lt. 7.2/Rt. −7.2	11.6	13.2	P1	19
TFL-D	Lt. −0.5/Rt. 0.5	−0.7	−19.0	P1	19
P1 total force	Lt. −64.7/Rt. 64.7	−15.2	80.7	P1	105
VL	Lt. −0.9/Rt. 0.9	−18.5	−92.9	P2	95
**Stair Climbing**					
**Force**	**X (N)**	**Y (N)**	**Z (N)**	**Acting Point**	**%**
Hip contact	Lt. 59.3/Rt. −59.3	60.6	−236.3	P0	251
ABD	Lt. 70.1/Rt. −70.1	28.8	84.9	P1	
ITT-P	Lt. 10.5/Rt. −10.5	3.0	12.8	P1	
ITT-D	Lt. −0.5/Rt. 0.5	−0.8	−16.8	P1	
TFL-P	Lt. 3.1/Rt. −3.1	4.9	2.9	P1	
TFL-D	Lt. −0.2/Rt. 0.2	−0.3	−6.5	P1	
P1 total force	Lt. −83.0/Rt. 83.0	−35.6	77.3	P1	119
VL	Lt. −2.2/Rt. 2.2	−22.4	−135.1	P2	137
VM	Lt. −8.8/Rt. 8.8	−39.6	−267.1	P3	270

ABD: abductor; ITT-P: iliotibial tract—proximal; ITT-D: iliotibial tract—distal; TFL: tensor fascia latae; TFL-D: tensor fascia latae—distal; TFL-P: tensor fascia latae—proximal; VL: vastus lateralis; VM: vastus medialis.

**Table 3:** Participant characteristics and details of the 3D-cTHA model.

Age [years]	66.5 ± 8.7
Height [m]	1.53 ± 0.06
Body weight [kg]	53.5 ± 9.0
Body mass index [kg/m^2^]	22.7 ± 3.2
Side [limb]	Left 16 Right 14
Bone mineral density of the femoral neck [g/cm^2^]	0.61 ± 0.13
Canal flare index	4.17 ± 0.42
Length of distal screw [mm]	25.7 ± 1.7
Femoral anteversion [degree]	21.69 ± 10.69

Data are presented as the mean ± SD.

**Table 4:** Stem-length-stratified maximum equivalent stress around the distal screw-removal holes on the medial and lateral sides.

	Medial		Lateral	
Stem Length (mm)	Maximum Equivalent Stress (MPa)	95% CI (MPa)	Maximum Equivalent Stress (MPa)	95% CI (MPa)
Normal walking				
130	79.630	(70.461–88.799)	58.500	(51.457–65.543)
140	77.970	(69.284–86.656)	55.640	(48.591–62.689)
150	73.330	(65.342–81.318)	53.310	(46.203–60.417)
160	73.235	(64.877–81.593)	52.900	(46.046–59.754)
Stair climbing				
130	124.017	(109.607–138.426)	72.887	(62.000–83.773)
140	122.635	(108.221–137.050)	71.427	(60.941–81.914)
150	115.587	(103.449–127.724)	67.537	(58.137–76.937)
160	115.147	(102.283–128.010)	67.330	(58.170–76.490)

## Data Availability

The data are available from the corresponding author if required.

## Abbreviations

The following abbreviations are used in this manuscript:

BMD	Bone mineral density
CFI	Canal Flare Index
cTHA	Conversion total hip arthroplasty
FEA	Finite element analysis
pTHA	Primary total hip arthroplasty
